# Elevated glutamine/glutamate ratio in cerebrospinal fluid of first episode and drug naive schizophrenic patients

**DOI:** 10.1186/1471-244X-5-6

**Published:** 2005-01-31

**Authors:** Kenji Hashimoto, Göran Engberg, Eiji Shimizu, Conny Nordin, Leif H Lindström, Masaomi Iyo

**Affiliations:** 1 Department of Psychiatry, Graduate School of Medicine, Chiba University, Chiba 260–8670, Japan; 2 Department of Physiology and Pharmacology, Karolinska Institute, 171 77 Stockholm, Sweden; 3 Department of Neuroscience and Locomotion, Division of Psychiatry, University Hospital, Linkoping, Sweden; 4 Department of Psychiatric Research, Västerås Central Hospital, University of Uppsala, Västerås, Sweden

## Abstract

**Background:**

Recent magnetic resonance spectroscopy (MRS) studies report that glutamine is altered in the brains of schizophrenic patients. There were also conflicting findings on glutamate in cerebrospinal fluid (CSF) of schizophrenic patients, and absent for glutamine. This study aims to clarify the question of glutamine and glutamate in CSF of first episode and drug naive schizophrenic patients.

**Method:**

Levels of glutamine and glutamate in CSF of 25 first episode and drug-naive male schizophrenic patients and 17 age-matched male healthy controls were measured by a high performance liquid chromatography.

**Results:**

The ratio (126.1 (median), 117.7 ± 27.4 (mean ± S.D.)) of glutamine to glutamate in the CSF of patients was significantly (z = -3.29, p = 0.001) higher than that (81.01 (median), 89.1 ± 22.5 (mean ± S.D.)) of normal controls although each level of glutamine and glutamate in patients was not different from that of normal controls.

**Conclusion:**

Our data suggests that a disfunction in glutamate-glutamine cycle in the brain may play a role in the pathophysiology of schizophrenia.

## Background

Multiple lines of evidence suggest that a dysfunction in glutamatergic neurotransmission might be involved in the pathophysiology of schizophrenia [1-6]. The amino acid glutamate plays a central role in nitrogen metabolism and participates in multiple biochemical pathways. Released glutamate is taken up by glia, where it is converted to glutamine, transported back to the presynaptic neuron, and reconverted to glutamate [6,7]. Thus, it seems that glutamate-glutamine cycle plays a role in the neuron-glia communication in the synapse, and that impairment of glutamate-glutamine cycle may be implicated in the pathophysiology of schizophrenia [1-6].

By means of *in vivo *proton magnetic resonance spectroscopy (MRS), a significant increase in glutamine level was detected in the medial prefrontal cortex of never-treated schizophrenic patients compared with controls [8]. In addition, a recent 4.0 T MRS study demonstrated that levels of glutamine in the left anterior cingulate cortex and thalamus of the never-treated patients with schizophrenia were significantly higher than those of healthy controls [9]. In contrast, significant lower levels of glutamine were found in the left anterior cingulate cortex of medicated patients with chronic schizophrenia than in the healthy controls, suggesting the role of chronic medication [10]. Thus, it is possible that the glutamate-glutamine cycle between neuron and glia may play a role in the glutamate hypothesis of schizophrenia.

Although Kim et al. [11] first reported reduction of cerebrospinal fluid (CSF) levels of glutamate in patients with schizophrenia, the findings of subsequent studies are inconsistent, with several report of no alteration in CSF levels of glutamate [12-14]. Furthermore, it was reported that a gradient for glutamate and glutamine in CSF was lack, and that there were significant correlations between the CSF and serum levels of glutamate (r = 0.67, p < 0.05) and glutamine (r = 0.84, p < 0.01)[15]. Moreover, sodium-dependent neutral amino acids transporters, located in the abluminal membranes of the blood brain barrier, are capable of actively removing neutral amino acids from the brain [16]. These findings suggest that concentration of neutral amino acids in the extracellular fluid of brain are maintained at ~10% of those of the blood [15,16].

In this study, we investigated whether levels of glutamate and glutamine or ratio of glutamine to glutamate in CSF of first episode and drug naive schizophrenic patients are different from those of age-matched healthy normal controls.

## Methods

Twenty-five male patients with schizophrenia (mean age 26.1 years, range 18–39) and 17 age-matched male healthy subjects (mean age 27.3 years, range 22–44) with no psychiatric disease were enrolled in Uppsala University and Linkoping University Hospital, Sweden. All patients diagnosed according to the DSM-III-R were first episode and drug naive, i.e. they had never been treated with antipsychotic drugs. In the morning (8:00–9:00) from May 1997 to January 1998, CSF of subjects was obtained by lumbar puncture (L4-L5), and twelve to eighteen μL of CSF was collected with a 0.9 mm needle and the samples were immediately frozen at -80°C, as reported previously [17]. The ethics committee of each institute approved the present study, and we received the informed consent from the participants of the study.

Measurement of glutamate and glutamine levels were carried out according to established methods [18] with a slight modification using a high performance liquid chromatography (HPLC) system with fluorescence detection (Shimadzu Corporation, Kyoto, Japan). Ten μL of the human CSF was added with 10 μL of 0.1 M borate buffer (pH 8.0) and 30 μL of 50 mM 4-fluoro-7-nitro-2,1,3-benzoxadiazole (NBD-F; Tokyo Kasei Kogyo Co., Ltd., Tokyo, Japan) in CH_3_CN. The reaction mixture was then heated at 60°C for 2 min, and immediately supplemented with 100 μL of H_2_O/CH_3_CN (90/10) containing 0.1 % trifluoroacetic acid (TFA) to stop the reaction. Ten μL of the resultant solution was injected into the HPLC system. A reversed-phase ODS column (TSKgel ODS-80Ts, Tosoh Corporation, Tokyo, Japan) was used for the separation and quantification of glutamate and glutamine, and the gradient elution of the mobile phase was kept at a constant flow rate of 0.8 mL/min. Mobile phase 1a consisted of H_2_O/CH_3_CN (90/10) containing 0.1 % TFA, and phases 1b and 1c, of H_2_O/CH_3_CN (10/90) containing 0.1 % TFA and CH_3_CN, respectively. The time program for gradient elution was programmed as follows: 0–50.5 min 1a: 1b : 1c = 95 : 5 : 0, 50.5–55.5 min 1a : 1b : 1c = 0 : 100 : 0, and 55.5–57 min, 1a : 1b : 1c = 0 : 0 : 100. The column temperature of all columns was maintained at 35°C. Fluorescence detection was made at 530 nm with an excitation wavelength at 470 nm.

Differences between two groups were analyzed using the Mann-Whitney U-test. The relationship between two variables was examined using Spearman correlation coefficients. A p < 0.05 was considered significant.

## Results

The CSF levels (421.7 μM (median), 468.1 ± 146.1 μM (mean ± S.D.), 254.0–775.1 (range)) of glutamine in the patients were not different (z = -1.038, p = 0.299) from those (410.5 μM (median), 405.6 ± 108.6 μM (mean ± S.D.), 219.8–689.0 (range)) of normal controls. The CSF levels (4.17 μM (median), 4.25 ± 1.77 μM (mean ± S.D.), 2.22–8.88 (range)) of glutamate in the patients were not different (z = -1.307, p = 0.191) from those (5.26 μM (median), 4.73 ± 1.29 μM (mean ± S.D.), 2.54–6.51 (range)) of normal controls. However, the ratio (126.1 (median), 117.7 ± 27.4 (mean ± S.D.), 42.0–161.6 (range)) of glutamine to glutamate in the CSF of patients was significantly (z = -3.29, p = 0.001) higher than that (81.01 (median), 89.1 ± 22.5 (mean ± S.D.), 59.7–134.0 (range)) of controls (Table 1). Furthermore, we found significant correlations between glutamate and glutamine in normal controls (r = 0.549, p = 0.022) or patients (r = 0.780, p < 0.001).

## Discussion

In this study, we found that the ratio of glutamine to glutamate in the CSF of first episode and drug naive schizophrenic patients was significantly higher than that of normal controls although each level of glutamine and glutamate in the CSF of patients was not significantly different from that of normal controls. To our knowledge, this is a first report demonstrating that the ratios of glutamine to glutamate in the first episode and drug naive patients are significantly higher than those of normal controls. In contrast, it was supposed earlier that alterations in CSF levels of glutamate are not so prominent compared with those in the brain [14]. Therefore, it is likely that a difference in glutamate (or glutamine) levels between our CSF study and MRS studies may be due to the difference between CSF samples and specific corticolimbic regions. However, it should be noted that alterations in the ratio of glutamine to glutamate are detected in the CSF samples of first episode and drug naive schizophrenic patients, suggesting an abnormality of the glia-neuronal glutamate-glutamine cycle in the brain of patients with schizophrenia.

In general, glutamine is synthesized in astrocytes from glutamate by the enzyme glutamine synthetase, found exclusively in brain glia cells. Glutamine then crosses the astrocytes to be transported into nerve cell terminals, where it is converted again into the neurotransmitter glutamate by glutaminase. It is reported that activities of glutaminase and glutamic acid decarboxylase (GAD; the rate-limiting enzyme in the synthesis of GABA by decarboxylation of glutamate) are significantly greater in the dorsolateral prefrontal cortex (DLPFC) of schizophrenia than in the control group, whereas activities of glutamate dehydrogenase, glutamine synthetase, and GABA transaminase in the DLPFC of schizophrenia are not different from the control group [19]. These findings suggest that metabolism of glutamate and GABA might be altered in the DLPFC of schizophrenic patients. Furthermore, it has been reported that activity of glutamine synthetase and glutamate dehydrogenase, the key enzymes involved in glutamate-glutamine cycle between neuron and glia, were markedly altered in the prefrontal cortex of schizophrenic patients, suggesting abnormalities in the function of glutamate-glutamine cycle in schizophrenic brain [20]. It is also well known that the glutamate-glutamine cycle between neuron and glia is tightly related to glutamate neurotransmission, glutamatergic function, and their regulation in human brain [7]. Taken together, it is likely that a dysfunction in glutamate-glutamine cycle in the brain may play a role in the pathophysiology of schizophrenia, supporting the glutamate hypothesis of schizophrenia.

As described in introduction, sodium-dependent amino acids transporters, located in the abluminal membranes of the blood brain barrier, are capable of actively removing amino acids from the brain [16,20,21]. Sodium-dependent amino acids transporter are capable of pumping both glutamine (system N) and glutamate (glutamate transporters EAAT-1, 2, and -3) from the extracellular fluid into endothelial cells [20,21]. The luminal facilitative carriers for both glutamate and glutamine can then transport them to the blood [16,20,21]. Therefore, the concentrations of naturally occurring amino acids in the CSF [presumably similar to the extracellular fluid of brain] are ~10% of those of the blood [15,16]. Taken together, it seems that alteration in the transport mechanisms regulating levels of glutamate and glutamine in CSF may be implicated in elevated glutamine/glutamate ratio in CSF of schizophrenic patients although further study is necessary.

## Conclusion

Our findings suggest that a dysfunction in glutamate-glutamine cycle between neuron and glia may play a role in the pathophysiology of schizophrenia, supporting the glutamate hypothesis of schizophrenia.

## Competing interests

The author(s) declare that they have no competing interests.

## Authors' contribution

KH conceived of the study, its design and coordination, and edited the manuscript. GE participated in the design of the study. CN and LHL recruited subjects and collected CSF samples. ES and MI assisted HPLC analysis and data analyses. All authors read and approved the final manuscript.

**Figure 1:**
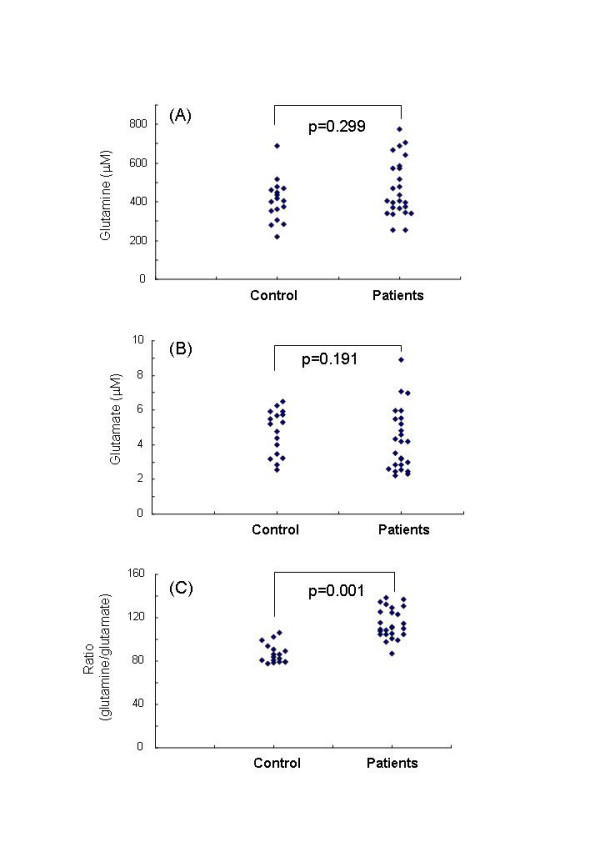
Levels of glutamine and glutamate, and ratio of glutamine to glutamate in CSF of normal controls, and first episode and drug naive schizophrenic patients. (A) CSF levels of glutamine in patients were not different from those of normal controls. (B) CSF levels of glutamate in patients were not different from those of normal controls. (C) Ratios of glutamine to glutamate in patients were significantly higher than those of normal controls.

## Pre-publication history

The pre-publication history for this paper can be accessed here:



## References

[B1] Javitt DC, Zukin SR (1991). Recent advances in the phencyclidine model of schizophrenia. Am J Psychiatry.

[B2] Olney JW, Farber NB (1995). Glutamate receptor dysfunction and schizophrenia. Arch Gen Psychiatry.

[B3] Goff DC, Coyle JT (2001). The emerging role of glutamate in the pathophysiology and treatment of schizophrenia. Am J Psychiatry.

[B4] Coyle JT, Schwarcz R (2000). Mind glue: implications of glial cell biology for psychiatry. Arch Gen Psychiatry.

[B5] Hashimoto K, Okamura N, Shimizu E, Iyo M (2004). Glutamate hypothesis of schizophrenia and approach for possible therapeutic drugs. Curr Med Chem CNS Agents.

[B6] Hashimoto K, Shimizu E, Iyo M (2005). Dysfunction of glia-neuron communication in pathophysiology of schizophrenia. Curr Psychiatry Rev.

[B7] Rothman DL, Behar KL, Hyder F, Shulman RG (2003). In vivo NMR studies of the glutamate neurotransmitter flux and neuroenergetics: implications for brain function. Annu Rev Physiol.

[B8] Bartha R, Williamson PC, Drost DJ, Malla A, Carr TJ, Cortese L, Canaran G, Rylett RJ, Neufeld RW (1997). Measurement of glutamate and glutamine in the medial prefrontal cortex of never-treated schizophrenic patients and healthy controls by proton magnetic resonance spectroscopy. Arch Gen Psychiatry.

[B9] Theberge J, Bartha R, Drost DJ, Menon RS, Malla A, Takhar J, Neufeld RW, Rogers J, Pavlosky W, Schaefer B, Densmore M, Al-Semaan Y, Williamson PC (2002). Glutamate and glutamine measured with 4.0 T proton MRS in never-treated patients with schizophrenia and healthy volunteers. Am J Psychiatry.

[B10] Theberge J, Al-Semaan Y, Williamson PC, Menon RS, Neufeld RW, Rajakumar N, Schaefer B, Densmore M, Drost DJ (2003). Glutamate and glutamine in the anterior cingulate and thalamus of medicated patients with chronic schizophrenia and healthy comparison subjects measured with 4.0-T proton MRS. Am J Psychiatry.

[B11] Kim JS, Kornhuber HH, Schmid-Burgk W, Holzmuller B (1980). Low cerebrospinal fluid glutamate in schizophrenic patients and a new hypothesis on schizophrenia. Neurosci Lett.

[B12] Perry TL (1982). Normal cerebrospinal fluid and brain glutamate levels in schizophrenia do not support the hypothesis of glutamatergic neuronal dysfunction. Neurosci Lett.

[B13] Do KQ, Lauer CJ, Schreiber W, Zollinger M, Gutteck-Amsler U, Cuenod M, Holsboer F (1995). γ-Glutamylglutamine and taurine concentrations are decreased in the cerebrospinal fluid of drug-naive patients with schizophrenic disorders. J Neurochem.

[B14] Tsai G, van Kammen DP, Chen S, Kelley ME, Grier A, Coyle JT (1998). Glutamatergic neurotransmission involves structural and clinical deficits of schizophrenia. Biol Psychiatry.

[B15] Alfredsson G, Wiesel FA, Lindberg M (1988). Glutamate and glutamine in cerebrospinal fluid and serum from healthy volunteers – analytical aspects. J Chromatogr.

[B16] O'Kane RL, Vina JR, Simpson I, Hawkins RA (2004). Na^+^-dependent neutral amino acid transporters A, ASC, and N of the blood-brain barrier: mechanisms for neutral amino acid removal. Am J Physiol Endocrinol Metab.

[B17] Erhardt S, Blennow K, Nordin C, Skogh E, Lindstrom LH, Engberg G (2001). Kynurenic acid levels are elevated in the cerebrospinal fluid of patients with schizophrenia. Neurosci Lett.

[B18] Aoyama C, Santa T, Tsunoda M, Fukushima T, Kitada C, Imai K (2004). A fully automated amino acid analyzer using NBD-F as a fluorescent derivatization reagent. Biomed Chromatography.

[B19] Gluck MR, Thomas RG, Davis KL, Haroutunian V (2002). Implications for altered glutamate and GABA metabolism in the dorsolateral prefrontal cortex of aged schizophrenic patients. Am J Psychiatry.

[B20] Burbaeva GSh, Boksha IS, Turishcheva MS, Vorobyeva EA, Savushkina OK, Tereshkina EB (2003). Glutamine synthetase and glutamate dehydrogenase in the prefrontal cortex of patients with schizophrenia. Prog Neuropsychopharmacol Biol Psychiatry.

[B21] Lee WJ, Hawkins RA, Vina JR, Peterson DR (1998). Glutamine transport by the blood-brain barrier: a possible mechanism for nitrogen removal. Am J Physiol.

[B22] O'Kane RL, Martinez-Lopez I, DeJoseph MR, Vina JR, Hawkins RA (1999). Na^+^-dependent glutamate transporters (EAAT1, EAAT2, and EAAT3) of the blood-brain barrier. A mechanism for glutamate removal. J Biol Chem.

